# Serum Proteome Profiling Identifies Novel and Powerful Markers of Cystic Fibrosis Liver Disease

**DOI:** 10.1371/journal.pone.0058955

**Published:** 2013-03-14

**Authors:** Timo Rath, Lisa Hage, Marion Kügler, Katrin Menendez Menendez, Reinhart Zachoval, Lutz Naehrlich, Richard Schulz, Martin Roderfeld, Elke Roeb

**Affiliations:** 1 Justus-Liebig-University Giessen, Department of Internal Medicine, Division of Gastroenterology, Giessen, Germany; 2 Ludwig-Maximilians-University Munich, Department of Gastroenterology, Munich, Germany; 3 Justus-Liebig-University Giessen, Department of Pediatrics, Division of Pulmonology, Giessen, Germany; 4 Justus-Liebig-University Giessen, Department of Internal Medicine, Division of Pulmonology, Giessen, Germany; University of Tübingen, Germany

## Abstract

**Background and Aims:**

Cystic Fibrosis associated liver disease (CFLD) develops in approximately 30% of CF patients. However, routine sensitive diagnostic tools for CFLD are lacking. Within this study, we aimed to identify new experimental biomarkers for the detection of CFLD.

**Methods:**

45 CF patients were included in the study and received transient elastography. Differential regulation of 220 different serum proteins was assessed in a subgroup of patients with and without CFLD. Most interesting candidate proteins were further quantified and validated by ELISA in the whole patient cohort. To assess a potential relation of biomarker expression to the degree of hepatic fibrosis, serum biomarkers were further determined in 18 HCV patients where liver histology was available.

**Results:**

43 serum proteins differed at least 2-fold in patients with CFLD compared to those without liver disease as identified in proteome profiling. In ELISA quantifications, TIMP-4 and Endoglin were significantly up-regulated in patients with CFLD as diagnosed by clinical guidelines or increased liver stiffness. Pentraxin-3 was significantly decreased in patients with CFLD. Serum TIMP-4 and Endoglin showed highest values in HCV patients with liver cirrhosis compared to those with fibrosis but without cirrhosis. At a cut-off value of 6.3 kPa, transient elastography compassed a very high diagnostic accuracy and specificity for the detection of CFLD. Among the biomarkers, TIMP-4 and Endoglin exhibited a high diagnostic accuracy for CFLD. Diagnostic sensitivities and negative predictive values were increased when elastography and TIMP-4 and Endoglin were combined for the detection of CFLD.

**Conclusions:**

Serum TIMP-4 and Endoglin are increased in CFLD and their expression correlates with hepatic staging. Determination of TIMP-4 and Endoglin together with transient elastography can increase the sensitivity for the non-invasive diagnosis of CFLD.

## Introduction

Cystic fibrosis associated liver disease (CFLD) has a cumulative incidence of approximately 30% [1], [2] and accounts for 2.5% of the overall mortality of CF patients, thereby representing the third most common cause of death in CF patients [3].

Due to the high prevalence, the early onset and the often progressive course of CFLD, the reliable recognition of CF patients at risk of developing CFLD is of urgent clinical necessity [4]. To date, liver histology is still the most commonly used gold standard for the assessment of chronic liver diseases. However, as only 1/50000 of the liver volume is evaluated with liver biopsy, it is controversially discussed in focally distributed liver disease and thereby not generally recommended for the assessment of CFLD [4], [5]. Further, liver biopsy exhibits a significant intra-/interobserver variability [4], [6], [7], [8] and, due to its invasive nature, is limited in its acceptance and repetition, especially in children [9], [10], [11]. Current guideline criteria recommend a combination of physical examination, liver biochemistry and ultrasound to diagnose CFLD [4]; however, the reliable identification of CF patients at risk of developing CFLD remains a major clinical challenge [4].

Consequently, research efforts have been focused on the development of non-invasive methods for the diagnosis of CFLD. First studies have successfully evaluated liver stiffness measured by transient elastography (TE) for the assessment of CFLD [12], [13], [14]. Additionally, a growing understanding of the pathogenesis of hepatic fibrosis identified non-invasive quantitative serum biomarkers of hepatic fibrogenesis, which are pathophysiologically derived from extracellular matrix (ECM) turnover and might directly translate the molecular pathogenesis of fibrosis into clinical application. Thereby, these so-called class I fibrosis markers, can act as potentially powerful serum biomarkers of hepatic fibrosis [14], [15], [16].

Within this study, we followed a dual intent: First, we aimed to identify novel serum biomarkers of liver disease in CF patients using a proteome profiling approach. Based on these results, we secondly verified increased expression of selected promising candidate proteins in CFLD by ELISA and further analysed their diagnostic value for the assessment of CFLD in comparison to that of TE. Using this approach, our results identify TIMP-4 and Endoglin as novel and promising serum markers of liver disease in CF patients that hold the potential to facilitate the non-invasive assessment of CFLD.

## Materials and Methods

### Patients

This study has been conducted according to the principles expressed in the Declaration of Helsinki. Written informed consent was obtained from all participating patients or their parents. The study was approved by the ethics committee of the medical faculty of the Justus-Liebig-University Giessen (Gaffkystrasse 11c, 35392 Giessen, Germany) with the approval no. 75/09. The diagnosis of CF was established by sweat test and later confirmed by genetic tests in all subjects. All patients were treated according to European and U.S. guidelines [17], [18]. After exclusion of other causes for chronic liver disease, the diagnosis of CFLD was established according to recent guidelines [4] if least two of the following conditions on at least two consecutive examinations spanning a one-year period were present: (i) Hepatomegaly (liver span >2 cm below the costal margin on the medioclavicular line) confirmed by ultrasound, (ii) two abnormal serum liver enzyme levels (ALT, AST, γGT > ULN), (iii) ultrasound abnormalities other than hepatomegaly (increased, heterogeneous echogenicity, nodularity, irregular margins).

To correlate the expression of experimental biomarkers to the histologic degree of hepatic fibrosis deposition, a cohort of 18 patients with chronic Hepatitis C Virus (HCV) infection was studied. In HCV patients, liver biopsy samples were taken via right intercostal space from the right liver lobe after desinfection and local anesthesia of the skin with an 18 gauge needle (Menghini needle, Braun-Melsungen, outer diameter 1.2 mm). An experienced pathologist who was blinded to the patients’ clinical results analyzed the biopsy specimens. Liver fibrosis stages were evaluated semi-quantitatively according to the Desmet/Scheuer [16] scoring system.

### Transient elastography (TE)

Liver stiffness by TE was evaluated using the same FibroScan® (Echosens, Paris, France) device in all patients. Non-invasive measurements were performed by a single experienced investigator blinded to the clinical status of the patients on the right lobe of the liver through the intercostal space at a depth of 25 and 65 mm from skin surface. In children below 15 kg of weight the FibroScan® S probe, developed for liver stiffness measurements in children, was used. For each patient, the stiffness value was calculated as the median of ten successful measurements. TE was considered valid if 10 successful measurements with a success rate ≥ 60% and an interquartile range ≤ 30% of the median were obtained. Results are expressed in kilopascal (kPa). Total examination time was approximately 5 minutes per patient.

### Routine laboratory tests and determination of biomarkers of hepatic fibrosis

All patients underwent routine haematological and biochemical investigations on the day of TE. The following proteome profiling arrays were used (all R&D Systems, Minneapolis, USA): human chemokine array kit (Cat. No. ARY017), human angiogenesis array kit (Cat. No. ARY007), human cytokine array panel A (Cat. No. ARY005), human soluble receptor array kit, non-hematopoietic panel (ARY012). Serum proteome profiler analyses for each array were at least repeated twice in two independent experiments and the volume of serum used ranged from 50 μl to 400 μl. TIMP-4, Endoglin, HGF and Pentraxin-3 were further quantified in undiluted serum (TIMP-4, Pentraxin-3) or in 1∶2 and 1∶10 diluted serum (HGF and Endoglin) with using commercially available ELISA Kits (Pentraxin-3: human Pentraxin-3 ELISA, BlueGene Biotech CO, Shanghai, China; TIMP-4: human TIMP-4 Quantikine ELISA Kit, Cat. No. DTM400; Endoglin: human Endoglin/CD105 Quantikine ELISA Kit, Cat. No. DNDG00; HGF: human HGF Quantikine ELISA Kit, Cat. No. DHG00, all R&D Systems, Minneapolis, USA).

### Statistical analysis

Statistical analysis was performed with SPSS 17.0 (SPSS Inc, Chicago, Ill). Normal distribution of the data was tested using the Kolmogorov–Smirnov test and visualization of histograms. Failing to meet criteria for normal distribution, differences in TE values and serum markers between patients with and without CFLD were assessed using the Mann-Whitney U test for unpaired samples. Liver stiffness and expression of serum markers are shown in Box-and-Whisker Plots. The upper and lower hinges of the box represent the 75th and 25th percentile, respectively. The line indicates the median value; error bars represent the minimum and maximum. Values deviating from the box by 1.5- to 3-fold interquartile range were defined as outliers (o). Significant differences are pointed out (*p<0.05, **p<0.01). Measurement agreement between clinical markers of CFLD and the novel diagnostic modalities assessed in the this studies has been performed in Bland-Altman-Analyses [19]. To directly compare the concordance of the differently scaled clinical and experimental markers every measured value “a” of the respective test was transferred into a commensurable variable “a′” using the following equation: a′ = (a- ā)/s_a_.

The diagnostic performances of TE and fibrosis markers were assessed by receiver operating characteristic (ROC) curves. The ROC curve is a plot of sensitivity versus (1 – specificity) for all possible cut-off values for the prediction of different fibrosis stages. The most commonly used index of accuracy is the area under the ROC (AUROC) curve with values close to 1.0 indicating a high diagnostic accuracy.

## Results

### Patient characteristics and proteome profile screening in patients with CFLD

From 45 CF patients that were consecutively enrolled between June 2008 and December 2010, 17 ( = 38%) were diagnosed with CFLD according to recent guidelines [4]. CF patients with CFLD had significantly elevated levels of liver transaminases and γ-GT whereas albumin levels and platelet counts were decreased in patients with CFLD compared to those without CFLD. Demographic and clinical characteristics of the CF study populations are shown in Table 1.

**Table 1:** Demographic and clinical data of the CF patient cohort.

	CF patients (n = 45)	
Characteristics	no CFLD (n = 28)	CFLD (n = 17)
**Demographic and clinical data**		
Male (n/%)	17/61%	9/53%
Female (n/%)	11/39%	8/47%
Age (y)		
mean (median) ± SD	21.4 (20.5) ± 11.8	29 (28) ± 10.8
range	5 – 50	14 – 47
BMI		
mean (median) ± SD	19 (18.9) ± 3.6	20.3 (20.3) ± 2.1
Pancreas insufficiency (n)		
no pancreatic insufficiency	5	0
pancreatic insufficiency	23	17
Treatment with UDCA (n)	9	15
**Biochemistry**		
Alanine aminotransferase (U/L)		
mean (median) ± SD	23 (22) ± 7.4	35 (33) ± 18 *
range	12 – 50	12 – 79
Aspartate aminotransferase (U/L)		
mean (median) ± SD	21 (20) ± 6.3	33 (27) ± 20.3 **
range	10 – 36	11 – 98
γ-glutamyl transpeptidase (U/L)		
mean (median) ± SD	14 (14) ± 6.5	86 (26) ± 110 **
range	6 – 34	9 – 321
Alkaline Phosphatase (U/L)		
mean (median) ± SD	179 (126) ± 106	248 (168) ± 185
range	62 – 452	84 – 668
Bilirubin (mg/dL)		
mean (median) ± SD	0.42 (0.4) ± 0.22	0.94 (0.68) ± 0.76
range	0.1 – 0.9	0.2 – 2.18
Albumin (g/dL)		
mean (median) ± SD	4.5 (4.5) ± 0.23	4.2 (4.1) ± 0.47 **
range	4.0 – 4.9	3.1 – 4.8
Prothrombin time (%)		
mean (median) ± SD	91 (95) ± 11	81 (84) ± 20.6
range	71 – 112	45 – 108
Platelet count (G/L)		
mean (median) ± SD	306 (316) ± 66	226 (277) ± 120 *
range	131 – 464	18 – 362

* significantly different, P <0.05

** significantly different, P <0.01

UDCA: Ursodeoxycholic Acid

**Figure 1:**
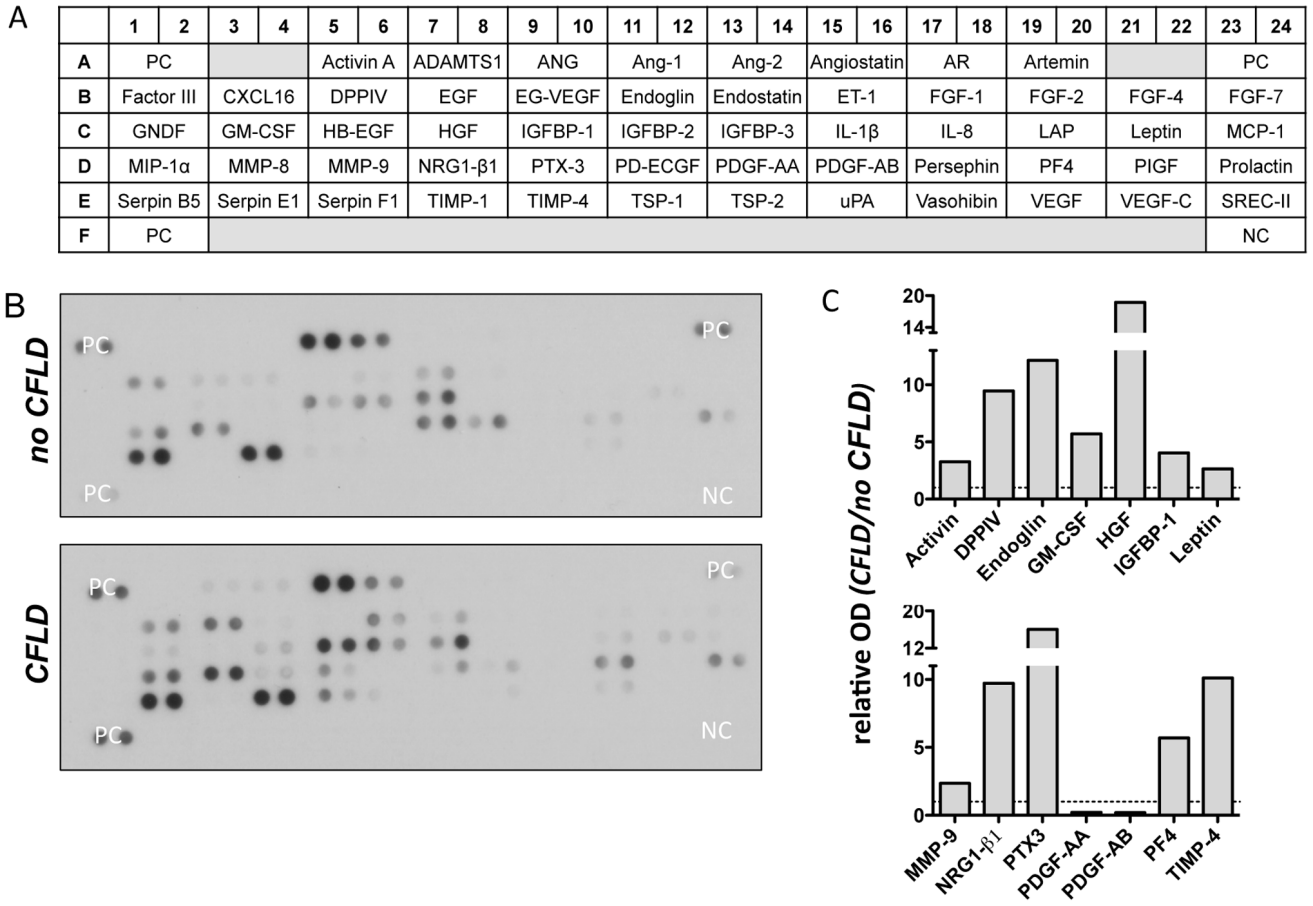
Assessment of angiogenesis related proteins in patients with CFLD. Relative expression of 55 different angiogenesis related proteins (A) were determined from pooled serum from patients with established CFLD (n = 4) and those without liver disease (n = 4). All proteins were determined in duplicate and based on optical densitometry of the corresponding bands (B), angiogenesis related proteins that were at least 2-fold differentially regulated in patients with CFLD compared to those without were identified. A total of 12 angiogenesis proteins were at least 2-fold increased in CFLD with highest relative expression observed for DDPIV, Endoglin, HGF, NRG1-β1, Pentraxin-3 and TIMP-4 (C). PDGF-AA and PDGF-BB were at least 2-fold decreased in patients with CFLD compared to those without (C). *PC: positive control, NC: negative control, OD: optical density*

**Figure 2:**
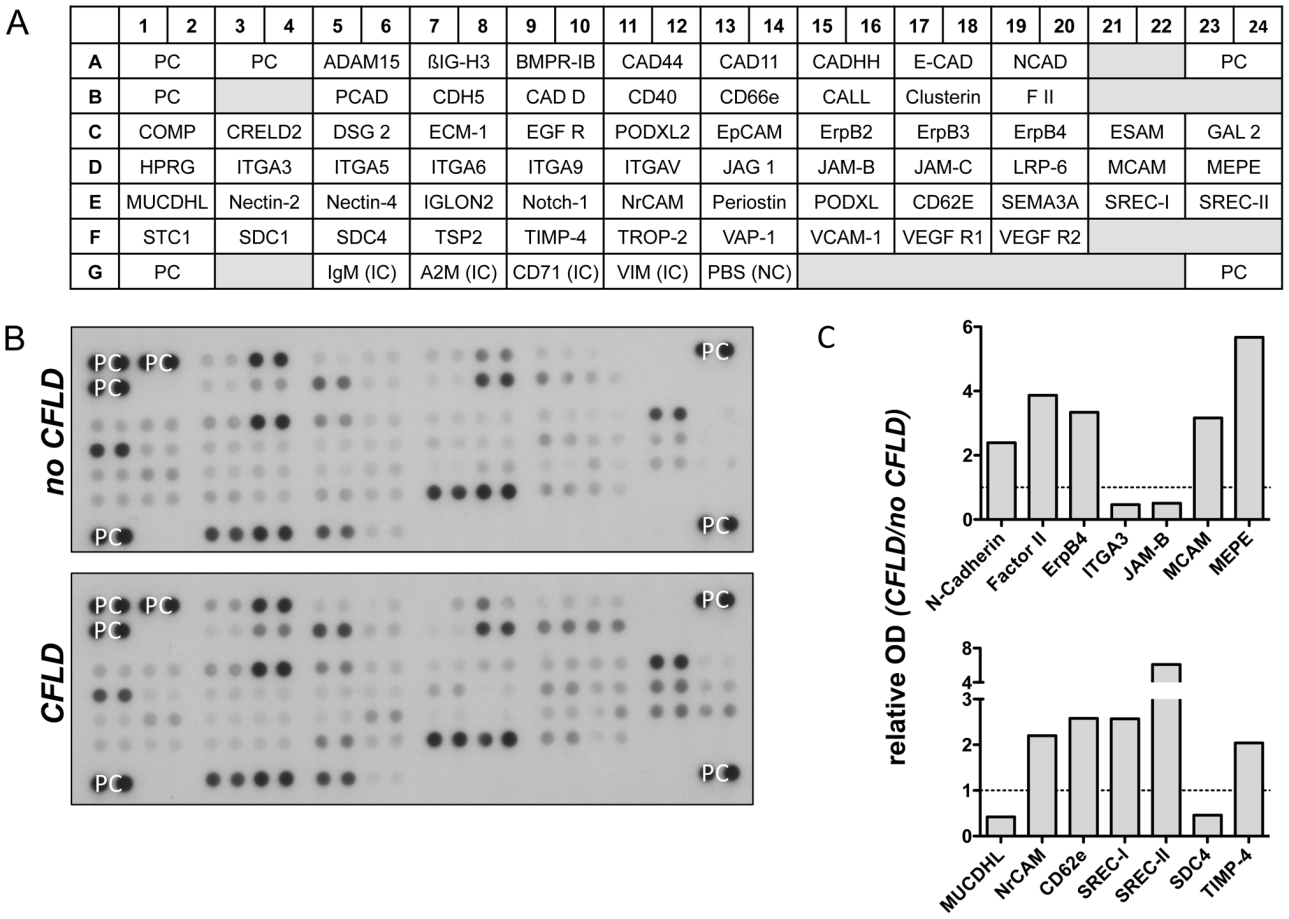
Assessment of soluble receptors and related proteins from non-hematopoietic cells in patients with CFLD (Array A). Relative expression of 62 different soluble receptors and related proteins from non-hematopoietic proteins (A) were determined from pooled sera from patients with established CFLD (n = 4) and those without liver disease (n = 4). All proteins were determined in duplicate and based on optical densitometry of the corresponding bands (B), proteins were identified that were at least 2-fold differentially regulated in patients with CFLD compared to those without. A total of 10 soluble receptors and related proteins from non-hematopoietic cells were at least 2-fold increased in CFLD with highest relative expression observed for MEPE and SREC-II (C). ITGA3, JAM-B, MUCDHL and SDC4 were at least 2-fold decreased in patients with CFLD compared to those without (C). *PC: positive control, NC: negative control, IC: internal control, OD: optical density*

**Figure 3:**
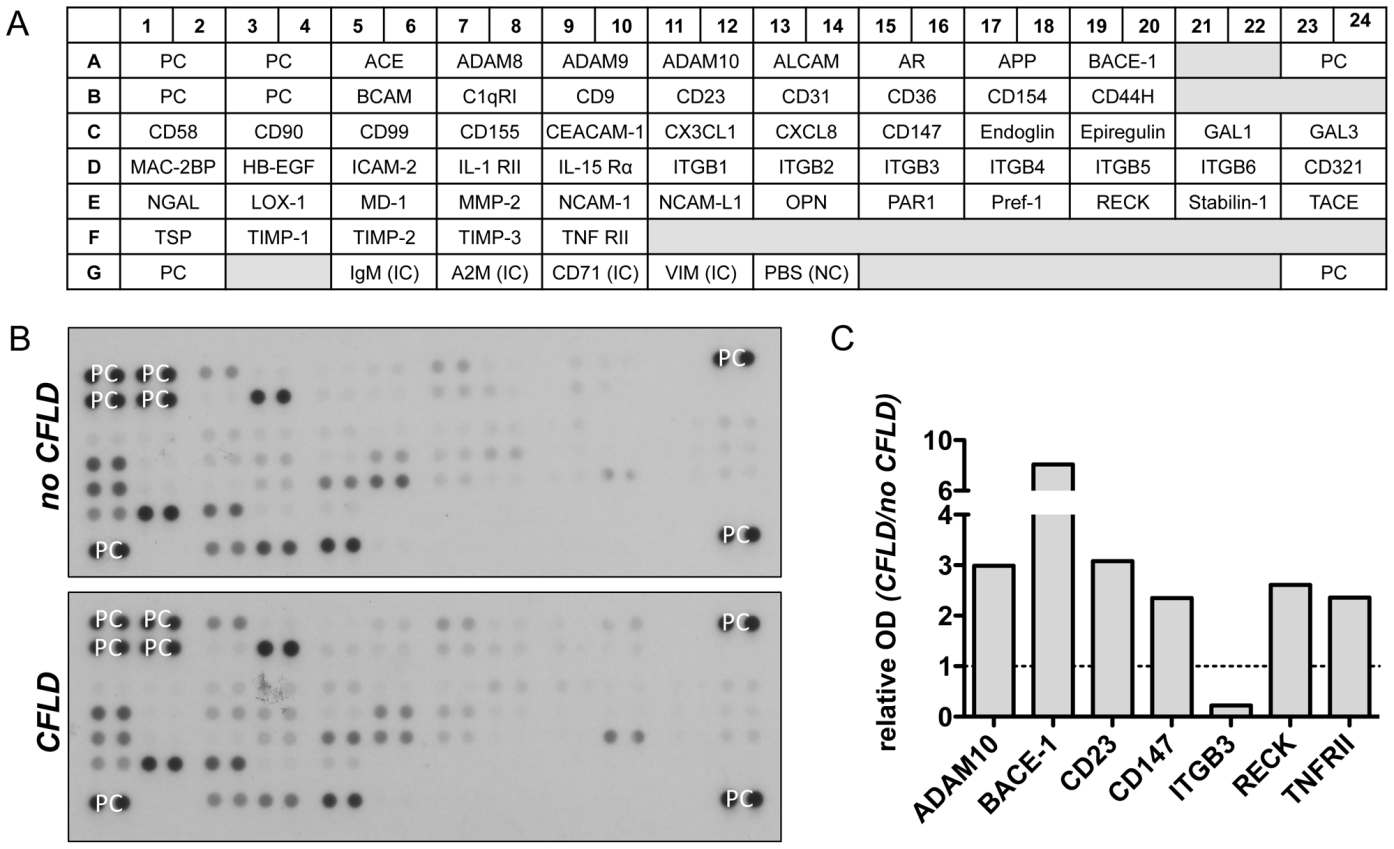
Assessment of soluble receptors and related proteins from non-hematopoietic cells in patients with CFLD (Array B). Relative expression of 57 different soluble receptors and related proteins from non-hematopoietic proteins (A) were determined from pooled serum from patients with established CFLD (n = 4) and those without liver disease (n = 4). All proteins were determined in duplicate and based on optical densitometry of the corresponding bands (B), proteins were identified that were at least 2-fold differentially regulated in patients with CFLD compared to those without. ADAM10, BACE-1, CD23, CD147, RECK, TNFRII were at least 2-fold increased in CFLD whereas ITGB3 was at least 2-fold decreased in patients with CFLD compared to those without (C). *PC: positive control, NC: negative control, IC: internal control, OD: optical density*

**Figure 4:**
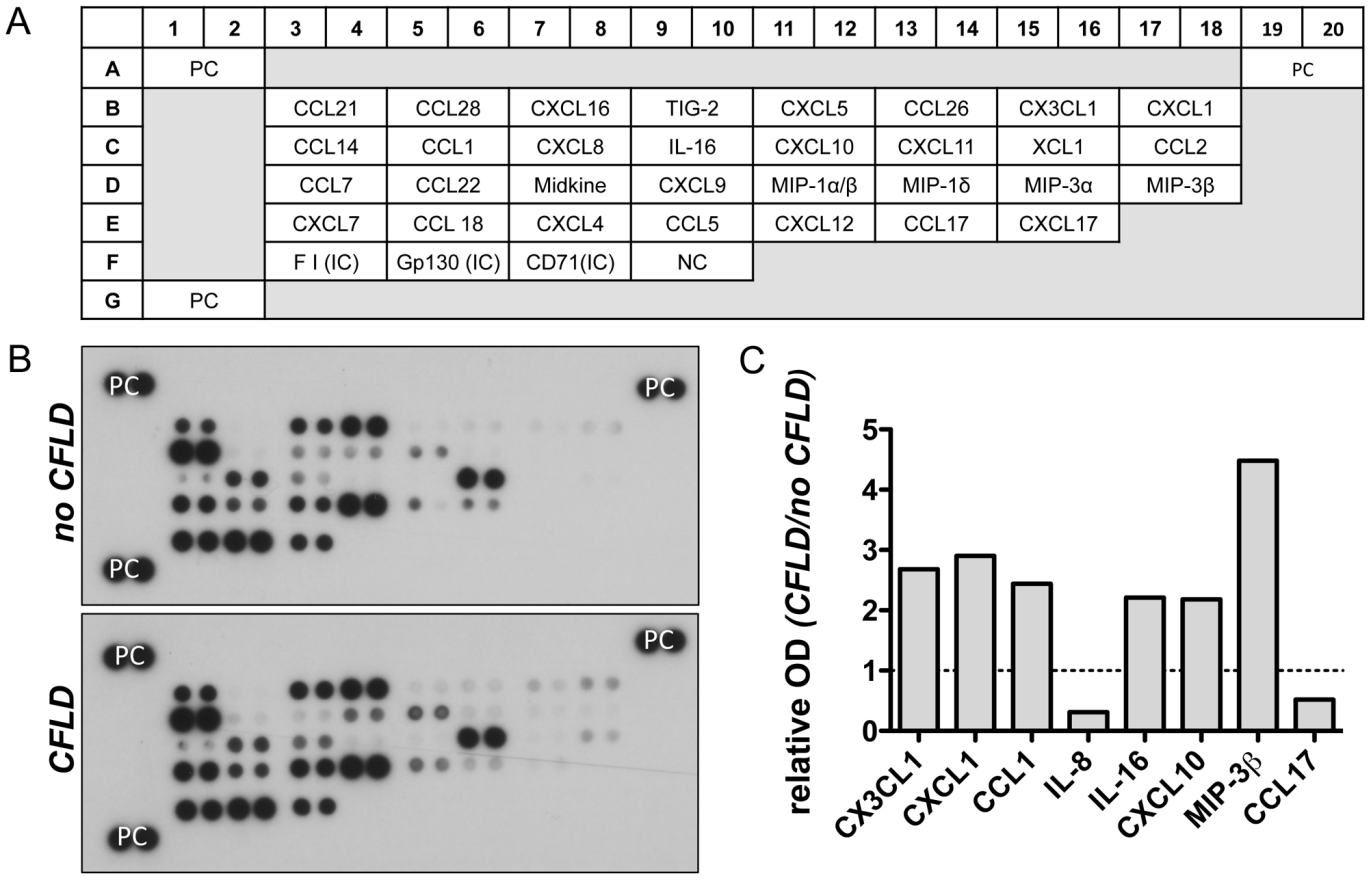
Assessment of chemokines in patients with CFLD. Relative expression of 31 different chemokines regulating the migration of monocytes, neutrophils, and lymphocytes (A) were determined from pooled serum from patients with established CFLD (n = 4) and those without liver disease (n = 4). All proteins were determined in duplicate and based on optical densitometry of the corresponding bands (B), proteins were identified that were at least 2-fold differentially regulated in patients with CFLD compared to those without. CX3CL1, CXCL1, CCL1, IL-16, CXCL10 and MIP-3β were at least 2-fold increased in CFLD whereas IL-8 and CCL17 was at least 2-fold decreased in patients with CFLD compared to those without (C). ). *PC: positive control, NC: negative control, IC: internal control, OD: optical density*

To identify potential candidate proteins which might act as biomarkers of hepatic fibrosis we initially screened a broad range of differentially regulated serum proteins in patients with CFLD and those without. For this purpose, serum of 4 patients with CFLD and of 4 patients without CFLD was applied to 4 different serum proteome profilers, thereby assessing key proteins and enzymes involved in angiogenesis (Figure 1), soluble receptors and related proteins released by non-hematopoietic cells and including key proteins involved in the metabolism of the extracellular matrix (Figure 2 and 3) and chemokines regulating the migration of monocytes, neutrophils, and lymphocytes (Figure 4). Serum proteome profiler analyses for each array were at least repeated twice in two independent experiments. Using this approach, we comparatively assessed the expression of a total of serum 220 proteins in patients without CFLD and those with proven CFLD. Based on optical densitometry analyses of the proteome profilers, 36 serum proteins were at least 2-fold increased in patients with CFLD compared to those without CFLD. Many of the proteins found up-regulated in CFLD have previously been described in fibrotic processes of different organs and/or liver fibrosis such as GM-CSF, ADAMTS-1, IP-10, PDGF-AB/-BB, TGF-ß1 or Activin A. Another 9 serum proteins were found to be at least 2-fold decreased in our serum proteome analysis.

Due to their relatively high abundance in CFLD patients and their pathophysiologic relation to the metabolism of extracellular matrix and/or angiogenesis as key events for the development of hepatic matrix deposition, we chose to further validate and quantify the serum levels of TIMP-4, Endoglin, Hepatocyte growth factor (HGF), and Pentraxin-3 (PTX3) in ELISA measurements in the whole CF cohort of 45 patients.

### Expression of serum fibrosis biomarkers in patients with CFLD

Serum was available from all 45 CF patients for quantification of the above-mentioned proteins by ELISA. All measurements were performed in duplicate.

**Figure 5:**
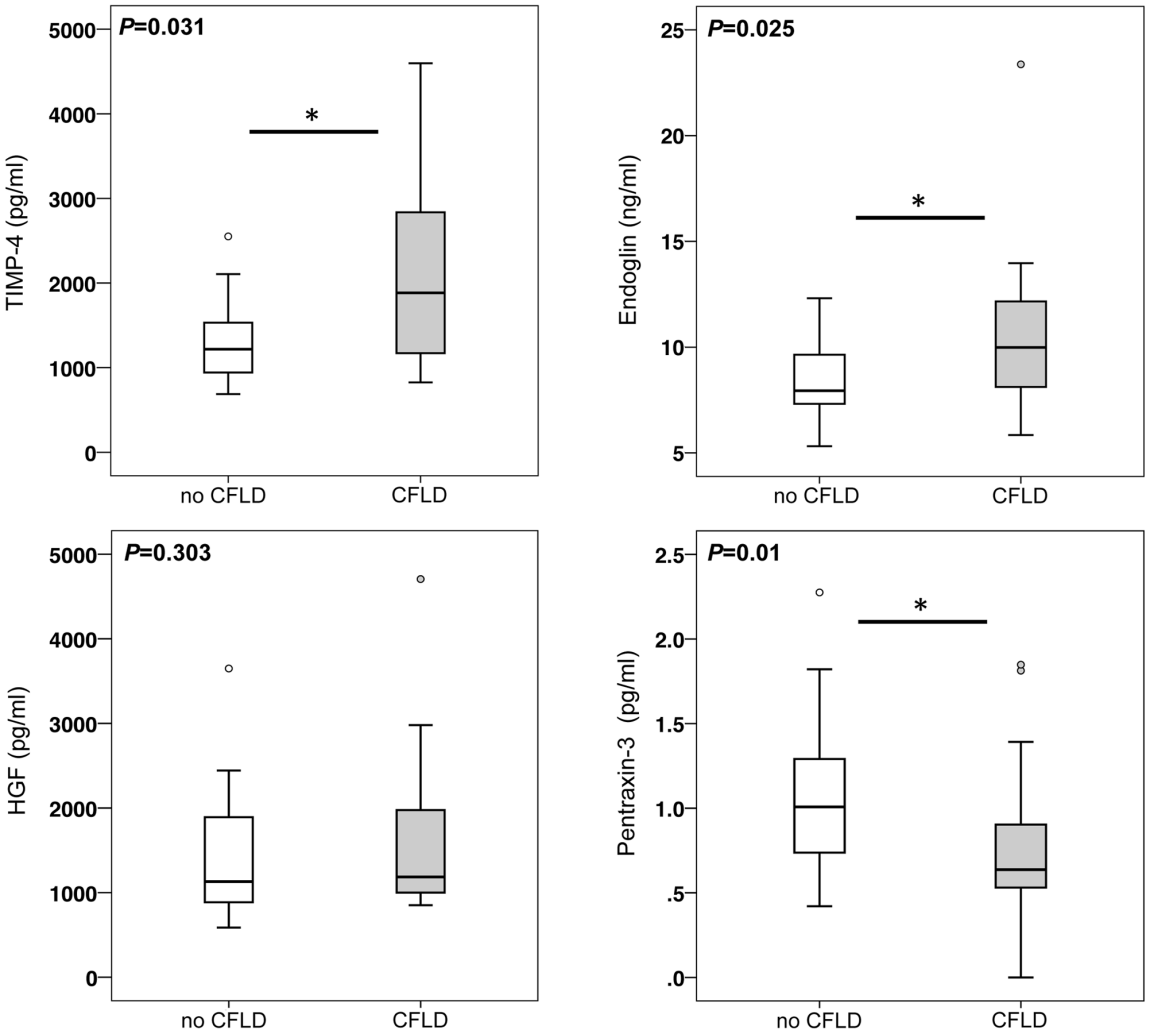
Increased serum concentrations of TIMP-4 and Endoglin and decreased levels of pentraxin-3 in CF patients with liver disease. In the whole CF cohort, patients with CFLD (n = 17) exhibited significantly higher serum levels of TIMP-4 and Endoglin than patients without liver disease (n = 28). Pentraxin-3 serum levels were significantly decreased in patients with CFLD whereas serum concentration of hepatocyte growth factor (HGF) was unchanged between CF patients with and without CFLD.

CF Patients with CFLD diagnosed according to recent guidelines exhibited significantly increased serum levels of TIMP-4 and Endoglin compared to those without liver disease. In contrast, serum PTX3 was significantly decreased in CF patients with hepatopathy. Serum levels of HGF did not significantly differ between CF patients with liver disease and those without (Figure 5).

**Figure 6:**
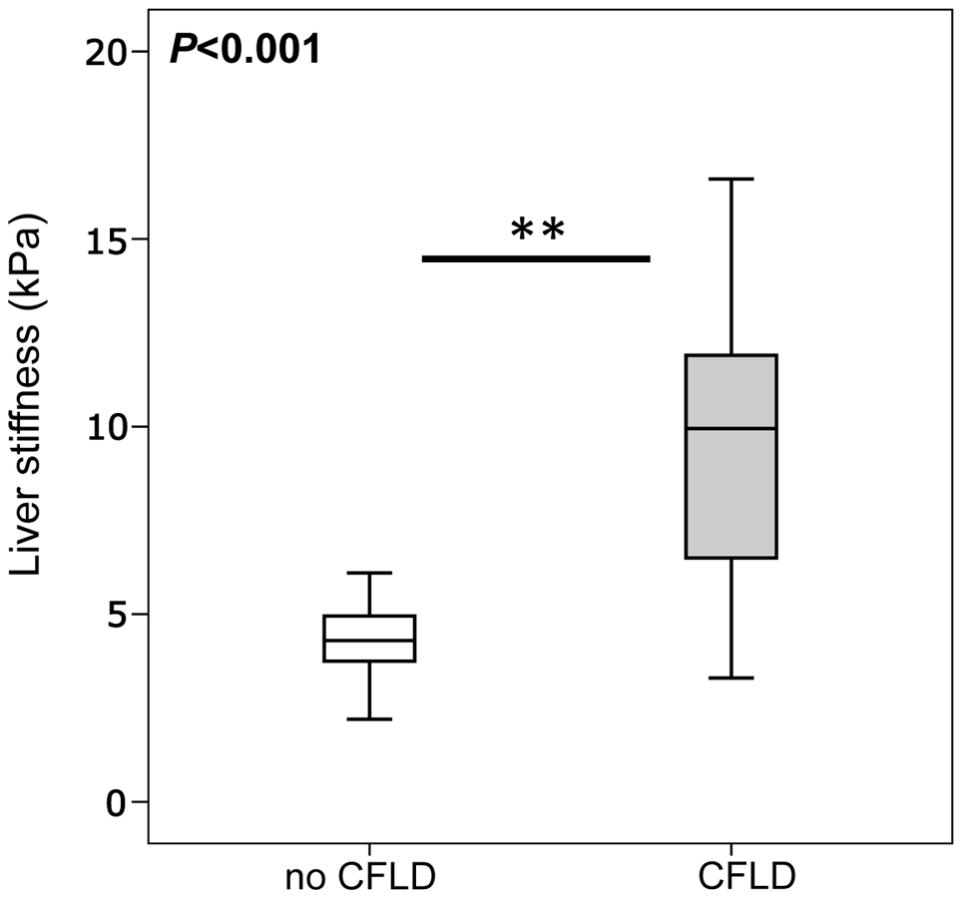
Increased liver stiffness in patients with CFLD. Median liver stiffness measured by transient elastography, was significantly increased in patients with CFLD (n = 17) compared to patients without liver disease (n = 28) (CFLD: 9.95 kPa vs no CFLD: 4.3 kPa). CFLD was diagnosed according to recent best practice guidelines [4].

As a further means for the assessment of the expression of serum fibrosis markers in CFLD, we measured liver stiffness values by transient elastography (TE) in our patient cohort, which has been proven to accurately diagnose liver disease and hepatic fibrosis of various etiologies, including CFLD [13], [14], [20], [21]. For this purpose we initially assessed the diagnostic value of TE in our CF patient cohort. As shown in Figure 6, with a median of 9.95 kPa, patients with CFLD exhibited a significantly higher liver stiffness compared to patients without liver disease, in which a median liver stiffness of 4.3 kPa was measured. Further, with an AUROC of 0.906, TE exhibited an excellent diagnostic accuracy in receiver operating characteristic (ROC) analysis and the sum of diagnostic sensitivity and specificity for the detection of CFLD was calculated to be maximal at a cut-off of 6.3 kPa based on ROC analysis (Table 2 and 3).

**Table 2:** Diagnostic accuracy of novel diagnostic markers and clinical markers for the detection of CFLD.

	ROC analysis		
	AUC	95% CI	*p-value*
**Novel diagnostic markers**			
Transient elastography	0.906	0.779 – 1.000	<0.001
TIMP-4	0.693	0.520 – 0.866	0.031
Endoglin	0.702	0.533 – 0.871	0.025
			
**Clinical markers**			
APRI	0.748	0.584 – 0.912	0.008
Alkaline phosphatase (ALP)	0.611	0.437 – 0.785	0.215

ROC: receiver operating characteristic; AUC: area under the curve; CI: confidence interval; APRI: AST/Platelets-Ratio-Index

**Table:** **Table 3.** Diagnostic performances of novel diagnostic markers and clinical markers for the detection of CFLD.

	Diagnostic performances					
	Cut-off		Sensitivity (%)	Specificity (%)	PPV (%)	NPV (%)
			(95% CI)	(95% CI)	(95% CI)	(95% CI)
***Clinical markers***						
APRI	0.133		46.7	96.4	87.5	77.1
			(22.3 – 72.6)	(79.8 – 99.8)	(46.7 – 99.3)	(59.4 – 89)
ALP	#		70.6	81.5	70.6	81.5
			(44 – 88.6)	(61.3 – 93)	(44 – 88.6)	(61.3 – 93)
***Novel markers***						
**- alone**						
TE	6.3 kPa		82	100	100	90.33
			(55.8 – 95.3)	(85 – 100)	(73.2 – 100)	(73.1 – 97.5)
TIMP-4	1603 pg/ml		64.7	82.1	68.8	79.3
			(38.6 – 84.7)	(62.4 – 93.2)	(41.5 – 87.9)	(59.7 – 91.3)
Endoglin	8.6 ng/ml		70.6	71.4	60	80
			(44 – 80.6)	(51.1 – 86)	(36.4 – 80)	(58.7 – 92.4)
***-*** ** in combination**						
TIMP-4 +	1603 pg/ml		88.2	53.6	53.6	88.2
Endoglin	8.6 ng/ml		(62.3 – 98)	(34.2 – 72)	(34.2 – 72)	(62.3 – 98)

TE +	6.3 kPa		88.2	71.4	65.2	90.9
Endoglin	8.6 ng/ml		(62.3 – 98)	(51.1 – 86)	(42.8 – 82.8)	(69.4 – 98.4)

TE +	6.3 kPa		100	82.1	77.3	100
TIMP-4	1603 pg/ml		(77.1 – 100)	(62.4 – 93.2)	(54.2 – 91.3)	(82.2 – 100)

# age and gender specific cut-off, values determined by the Department for Laboratory Medicine and Clinical Chemistry of the University Hospital Giessen according to the International Federation of Clinical Chemistry

PPV: positive predictive value; NPV: negative predictive value; CI: confidence interval

**Figure 7:**
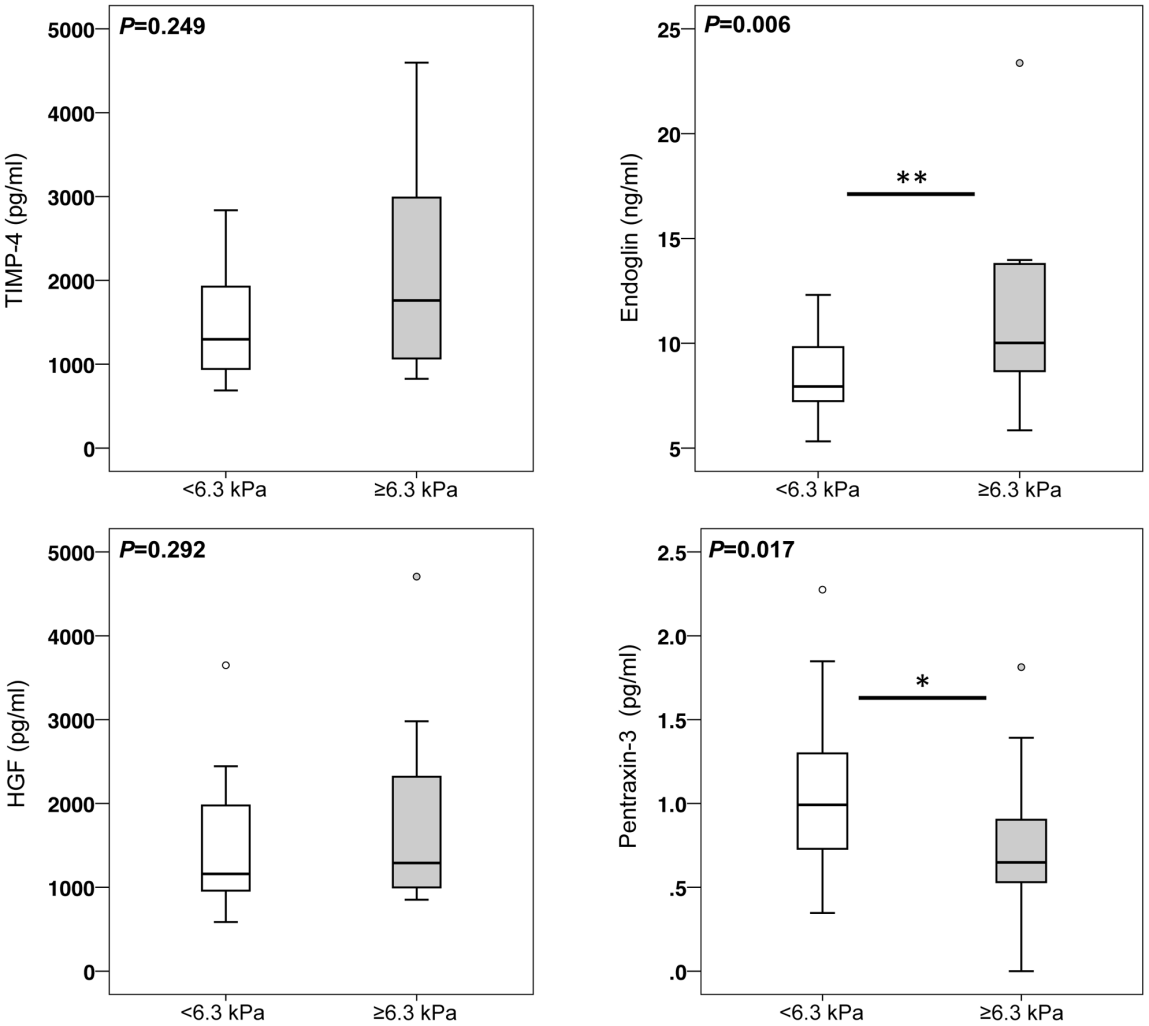
Increased serum levels of Endoglin and decreased concentration of pentraxin-3 in CF patients with increased liver stiffness. CF patients with liver stiffness values above a threshold value of 6.3 kPa, indicative for CFLD, exhibited significantly higher serum levels of Endoglin and significantly lower serum levels of pentraxin-3 compared to CF patients with liver stiffness values below 6.3 kPa. TIMP-4 was also increased in patients with a liver stiffness above the threshold of 6.3 kPa, although these elevations did not reach statistical significance. The cut-off value of 6.3 kPa for the identification of patients with CFLD was derived from ROC analyses as value at which the sum of diagnostic sensitivity and specificity for the detection of CFLD was maximal (Table 3).

When serum levels of hepatic fibrosis markers were then compared in patients with TE values below or above this threshold, we observed similar results to those obtained from the comparison of patients with and without CFLD as defined by recent guidelines: Endoglin was significantly increased in patients with liver stiffness values above 6.3 kPa whereas PTX3 was again significantly decreased in patients with TE values above 6.3 kPa (Figure 7). As seen in patients with CFLD as diagnosed by guideline criteria, TIMP-4 serum levels were also increased in patients with stiffness values >6.3 kPa compared to those with values below 6.3 kPa, although these differences did not reach statistical significance. Serum expression of HGF was unchanged between patient with and without CFLD, as diagnosed by TE with a cut-off of 6.3 kPa (Figure 7).

To address CF lung manifestation and pulmonary fibrosis as a potential confounder of the above results of a significant regulation of TIMP-4, Endoglin and PTX3 in the presence of liver disease, we assessed their serum expression in CF patients with a forced expiratory volume in one second (FEV1) below and above 70%, with a vital capacity (VC) of below and above 80%, and with a ratio between FEV1 and VC below and above 70% (FEV1/VC), which serve as established indicators of CF lung disease and have been the primary outcome in many clinical trials [17], [22], [23], [24]. Of note, none of the above serum markers was different in patients with and without impairment of lung function as assessed by FEV1, VC and FEV1/VC (Figure S1). Further, serum marker levels were unaltered between patients without pancreatic insufficiency (PI, Figure S2), indicating that the increased expression of TIMP-4 and Endoglin and the decreased expression of PTX3 occur indeed relatively specific for the existence of liver disease without being affected by pancreas and lung disease as other major manifestations of CF.

As treatment with Ursodeoxycholic acid (UDCA) improves liver function tests and biliary drainage and thus represents the guideline recommended therapy for CFLD [4], we further assessed whether liver stiffness and biomarker expression was influenced by the treatment with UDCA. While TE values were slightly increased in patients that received UDCA, the biomarkers TIMP-4, Endoglin and PTX3 were not significantly altered between CF patients with and without UDCA treatment (Figure S3) and there was no association between the duration of UDCA therapy and liver stiffness and biomarker expression (data not shown).

### Expression of serum markers of hepatic fibrosis correlates with hepatic staging

**Figure 8:**
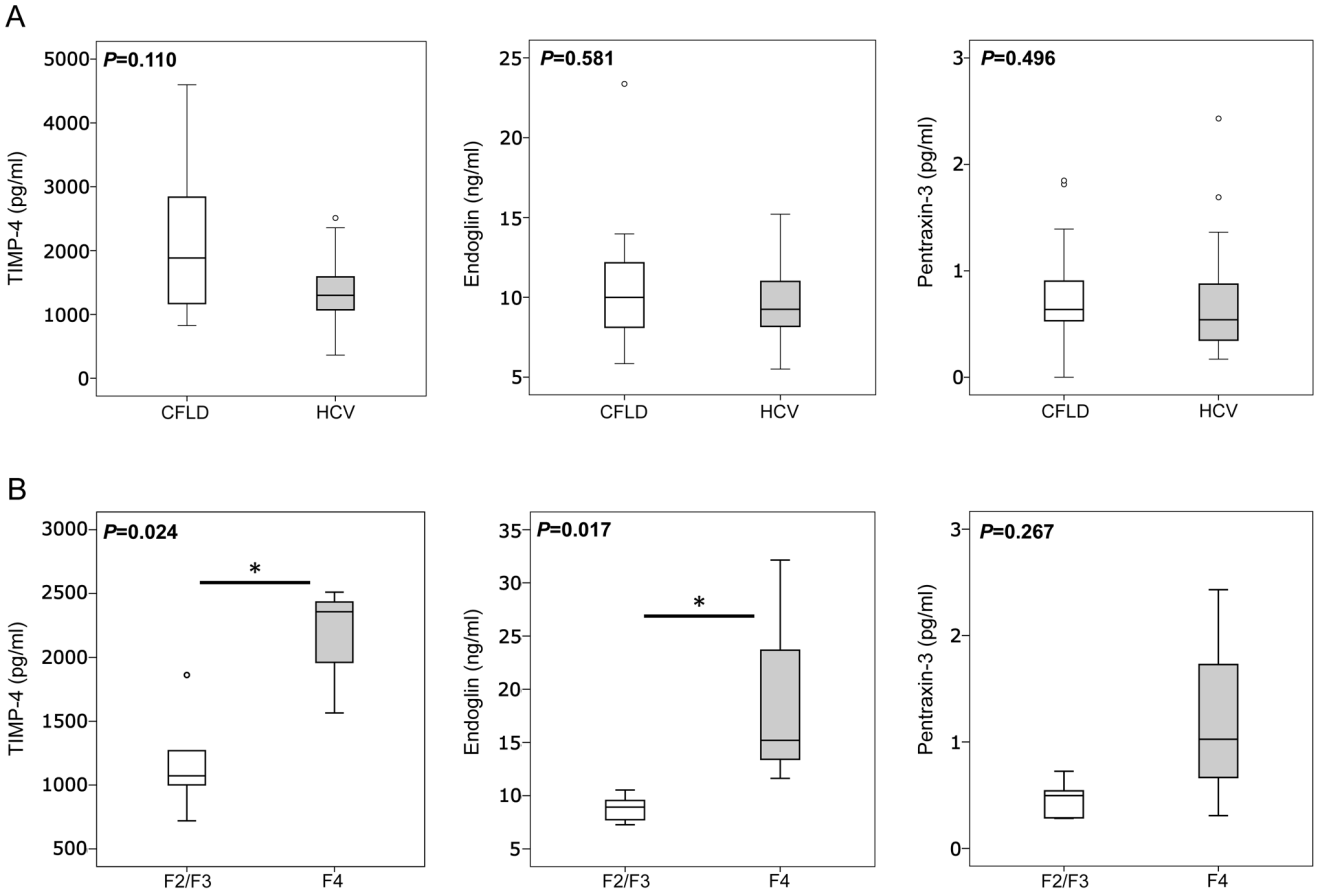
Increased serum levels of TIMP-4 and Endoglin in HCV patients with complete liver cirrhosis. Serum levels of TIMP-4, Endoglin and Pentraxin-3 were comparable and without significant differences between patients with CFLD and those with HCV liver disease (top row, A). In the cohort of patients with HCV liver disease, patients with complete fibrosis/cirrhosis (F = 4) exhibited significantly increased serum levels of TIMP-4 and Endoglin compared those HCV patients with portal fibrosis with few or numerous fibrotic septae but without cirrhosis (F = 2/3), whereas Pentraxin-3 remained unchanged in the different HCV fibrosis stages (bottom row, B).

Based on the above results we hypothesized that serological expression of TIMP-4, Endoglin and Pentraxin-3 might correlate with hepatic staging as determined by liver histology. However, due to the high percentage of children (35.6%) in our CF patient cohort and as liver staging by histology has further been controversially discussed or even to be unreliable in focally distributed liver disease as seen in CFLD, we did not assess liver histology in our CF patients. We instead assessed serum expression of TIMP-4, Endoglin and Pentraxin-3 in 18 patients with HCV that underwent liver histology during routine clinical care. Interestingly, serum levels of all three biomarkers were comparable and without significant differences between patients with CFLD and patients with HCV liver disease (Figure 8A). When serum levels of TIMP-4, Endoglin and Pentraxin-3 were then compared between HCV patients with complete fibrosis/cirrhosis (F = 4) and those HCV with portal fibrosis with few or numerous fibrotic septae but without cirrhosis (F = 2/3), significantly increased levels of TIMP-4 and Endoglin in HCV patients with complete fibrosis/cirrhosis were found. Pentraxin-3 remained unchanged in the different HCV fibrosis stages (Figure 8B). Taken together, these results suggest that increased expression of TIMP-4 and Endoglin represents a mechanism specific for hepatic fibrosis that correlates with hepatic staging but is irrespective of the underlying etiology of liver disease.

### Diagnostic performances of biomarkers of hepatic fibrosis and transient elastography for the detection of CFLD

**Figure 9:**
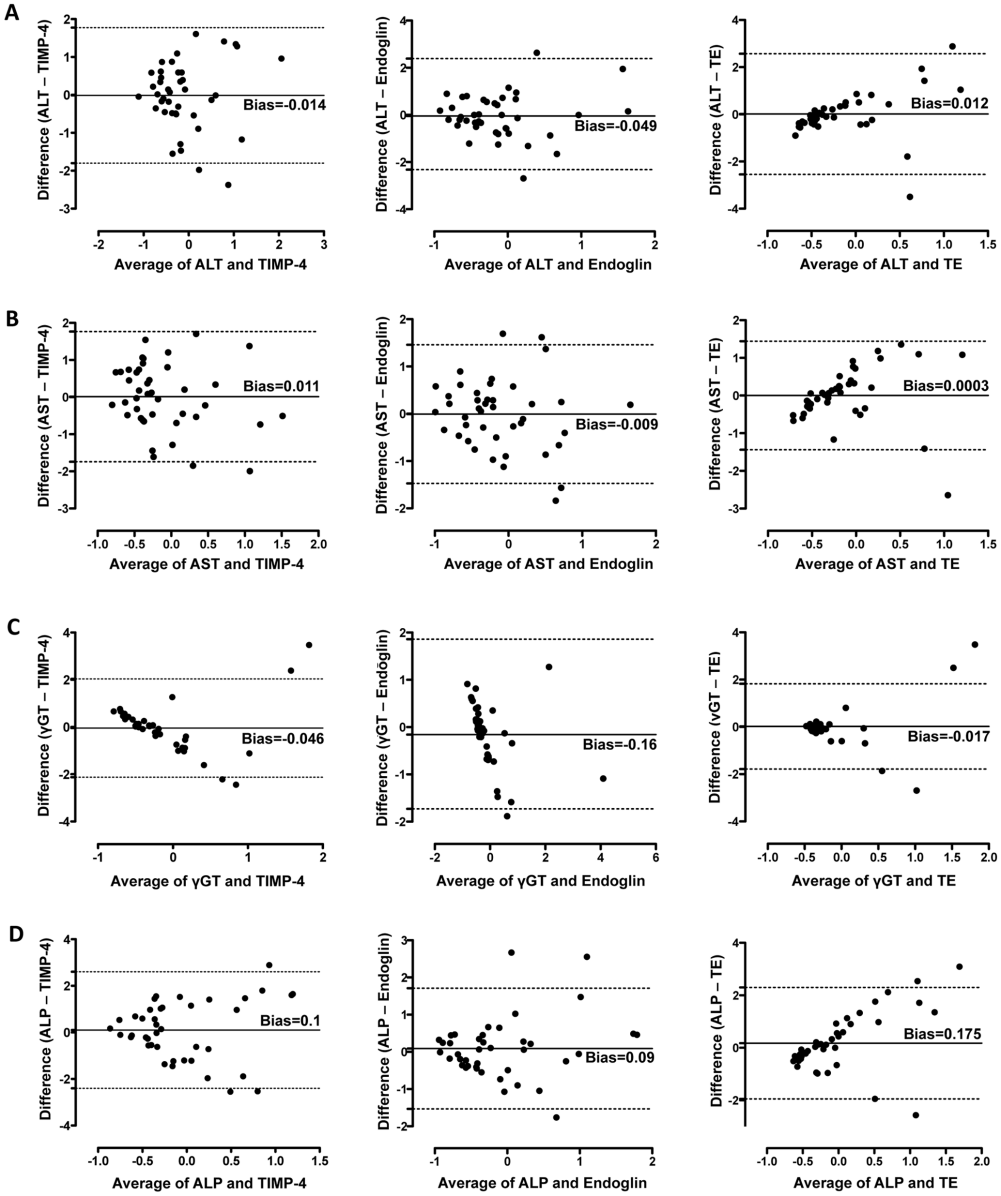
Concordance between clinical markers and novel diagnostic markers for CFLD. Measurement agreement between AST, ALT, γGT and ALP as commonly used clinical markers of CFLD and TIMP-4, Endoglin and transient elastography as novel diagnostic modalities was assessed in Bland-Altman Analyses. TIMP-4 and Endoglin showed very good agreement with ALT (A) and AST (B) levels. Comparison of the concordance of TE and levels of ALT (A), AST (B) and γGT (C) showed good agreement of the different methods although a greater bias was observed with higher average values. A proportional error was observed in the agreement of γGT and the biomarkers TIMP-4 and Endoglin (C) and in the agreement of ALP and TE (D). The dashed line shows 95% limits of agreement, the solid line indicates the systemic error (bias).

Based on these results, we hypothesized that the observed increased expression of TIMP-4 and Endoglin in CFLD might well be usable in diagnostic approaches. To provide an outlook on a potential diagnostic utility of TIMP-4, Endoglin and TE for CFLD, we first compared the agreement of TIMP-4, Endoglin and TE measurements to levels of ALT, AST, γGT and ALP as commonly used and established parameters for CFLD diagnostic in Bland-Altman Analyses. As shown in Figure 9, TIMP-4 and Endoglin showed very good agreement with ALT (Figure 9A) and AST (Figure 9B) levels. Comparison of the concordance of TE and levels of ALT (Figure 9A), AST (Figure 9B) and γGT (Figure 9C) showed good agreement of the different methods likewise; however, a greater bias was observed with higher average values. Further, when clinical cholestasis markers were compared with the novel modalities for CFLD diagnosis assessed in this report, Bland-Altman Analyses revealed the presence of a proportional error in the agreement of γGT and the biomarkers TIMP-4 and Endoglin (Figure 9C) and in the agreement of ALP and TE (Figure 9D).

We then aimed to directly compare the diagnostic performance of TIMP-4, Endoglin and TE as novel diagnostic markers for CFLD to clinical markers of hepatopathy. Importantly, the liver enzymes AST, ALT and γGT were part of the diagnostic algorithm used for identification of patients with CFLD in this study, thereby making it impossible to assess their diagnostic performance accurately and objectively within this study. To counteract this potential selection bias of AST, ALT and γGT in their assessment of the diagnostic value of common clinical markers, we instead calculated the diagnostic performances of the AST/Platelets-Ratio-Index (APRI) as a non-invasive fibrosis index [25], [26] and that of alkaline phosphatase, which also has not been part of the diagnostic algorithm to diagnose CFLD. As assessed in ROC analyses, with and AUROC of 0.908 TE exhibited an excellent accuracy for the detection of CFLD. Further, with an AUROC of 0.702 and 0.693, Endoglin and TIMP-4 exhibited a good accuracy for CFLD diagnosis (Table 2). Importantly, TE exhibited a by far superior diagnostic accuracy for CLFD compared to APRI and ALP whereas the biomarkers Endoglin and TIMP-4 exhibited a better (AP) or almost equal diagnostic accuracy (APRI) when compared to the clinical markers (Table 2).

At a cut-off of 8.6 ng/ml for Endoglin and 1603 pg/ml for TIMP-4, the sum of sensitivity and specificity were calculated to be maximal for the detection of CFLD (Table 3).

Importantly, when Endoglin or TIMP-4 at these cut-off values were then combined with TE for the diagnosis of CFLD, the diagnostic sensitivity of TE improved considerably: for TE alone, a diagnostic sensitivity of 82% was calculated; when TE was combined with TIMP-4 or Endoglin, the sensitivity improved to 100% and 88%, respectively. As a result, the negative predicative value was also considerably improved when TE was combined with TIMP-4 and Endoglin compared to TE alone. The diagnostic performances of TE, Endoglin, and TIMP-4 alone or combined and in comparison to the clinical markers ALP and APRI are shown in Table 3.

## Discussion

Currently established approaches used for diagnosis and follow-up of CFLD exhibit certain limitations such low sensitivity (liver enzymes, ultrasound), invasiveness and sampling error (biopsy), exposure to radiation (CT scan) or high costs (MRI) [27].

However, as CFLD occurs in approximately 30% of all CF patients [1], [2], [28] and in 5 to 10% of CF patients progression towards multilobular cirrhosis occurs early in life [1], [2], reliable non-invasive diagnostic tests for CFLD are urgently needed. Regarding the special requirements in children, non-invasive tools should also enable a fast but still accurate CFLD screening in daily practice and allow for cost-effective longitudinal follow-up.

With the intention to identify novel candidate protein acting as serum markers of liver disease, we initially screened a broad range of serum protein in patients with and without CFLD using a proteome profiling approach. Based on these results we then quantified expression of TIMP-4, Endoglin, HGF and Pentraxin-3 as promising and novel serum markers in a larger cohort of CF patients and comparatively analysed their diagnostic value for the detection of CFLD compared to that of TE. Our results provide evidence (i) that serum TIMP-4 and Endoglin are significantly increased in patients with CFLD compared to those without while serum Pentraxin-3 levels are significantly decreased in CFLD patients; (ii) that TIMP-4 and Endoglin show highest expression in patients with complete liver fibrosis/cirrhosis as exemplified in HCV patients (iii) that TIMP-4 and Endoglin exhibit a good diagnostic accuracy for the detection of CFLD; (iv) that the determination of serum TIMP-4 and Endoglin together with transient elastography can increase the diagnostic sensitivity for the non-invasive detection of CFLD.

Our results of elevated liver stiffness in patients with CFLD corroborate the results of previous studies. In a cohort of pediatric patients with liver disease of various etiologies, among them 20 patients with CF, Breton and co-workers showed that patients with liver disease had significantly increased liver stiffness values [29]. Specifically, in CFLD, Witters and co-workers recently demonstrated that adults and children with CFLD had a significantly elevated mean liver stiffness of 8.8 kPa compared to 5 kPa in individuals without CFLD [13]. In a very recent report, it was further shown that CF patients with CFLD had increased TE values compared to patients without CFLD, with highest liver stiffness measurements seen in patients with cirrhosis [21]. Interestingly, with a mean liver stiffness of 9.95 kPa in patients with CFLD compared to 4.3 kPa in those without CFLD, we found very similar data in our total patient cohort compared to those described by Witters and colleagues. Furthermore, a cut-off of 6.3 kDa was calculated to be optimal for the detection of CFLD within the present study, thereby corroborating the cut-off defined in previous studies on the detection of CFLD by TE [14], [21].

Apart from TE, research has identified potentially powerful quantitative serum biomarkers of hepatic fibrosis [14], [15], [16]. Above all, so called class I markers which are pathophysiological derived from the metabolism of the extracellular matrix, directly translate the molecular pathogenesis of fibrosis into clinical application and therefore represent promising fibrosis markers [15]. Generally spoken, an ideal marker for liver fibrosis should have a high sensitivity and specificity, and should also be easily assessable inexpensive, accurate, and reproducible for follow-up [30]. Serum fibrosis markers are easily obtained and, unlike special diagnostic procedures such as transient elastography that is mainly limited to larger clinics and centers, are widely available and therefore represent and an appealing possibility to fulfill these outlined criteria. Given these considerations, we chose to screen a broad spectrum of candidate proteins that might serve as serum markers of liver disease and hepatic fibrosis in CFLD patients using an unbiased proteome profiling approach. We simultaneously determined the relative expression of a total of 220 serum proteins in patients with CFLD and those without. Our results show that 36 serum proteins are at least 2-fold increased in patients with CFLD. Among these up-regulated proteins, we found several proteins that have been well described in the context of hepatic fibrosis, e.g. such as LAP/TGF-ß1 [31], [32], members of the activin family [33], the plasminogen activator system such as uPA [34], or members of the CX_3_C chemokine family such as fractalkine [35]. Apart from that, our study identified further proteins previously not been described in the context of liver disease and fibrosis. Based on their relatively high abundance in the proteome screen and there close pathophysiologic relation to the metabolism of extracellular matrix and/or angiogenesis as key events for the development of hepatic matrix deposition, we chose to further validate and quantify the serum levels of TIMP-4, Endoglin, Hepatocyte growth factor (HGF), and Pentraxin-3 (PTX3) in ELISA measurements in the whole CF cohort of 45 patients.

Tissue inhibitors of metalloproteinases (TIMPs) have been shown to be critically involved in hepatic fibrogenesis. Their fibrogenetic potential is believed to be mediated by the inhibition of ECM degradation and the subsequent accumulation of fibrotic tissue [36]. Enhanced expression of TIMP-1 and TIMP-2 occurs in rat models of liver injury [37] and in various human liver diseases [38]. In patients with chronic HCV infection, TIMP-1 mRNA levels correlate with the grade of liver fibrosis [39] and HCV itself is able to stimulate TIMP-1 mRNA expression [40]. Serum TIMP-2 and liver TIMP-2 mRNA are also increased in HCV induced liver disease [40], [41]. TIMP-4 mRNA has been shown to be increased in a model of experimental biliary atresia [42], but apart from that data on the role of TIMP-4 for liver disease and hepatic fibrosis are scarce. Due to their prominent role in hepatic fibrogenesis, members of the TIMP-family have been utilized in diagnostic panels and indices [43], [44]. In this report, we now show that TIMP-4 as a previously rather unrecognized member of the TIMP-family is significantly increased in cystic fibrosis associated liver disease. As a proof of principle in HCV patients, we also provide evidence that TIMP-4 serum levels show highest values in patients with complete liver cirrhosis. Further, in the CF patient cohort TIMP-4 exhibited a high accuracy for the detection of liver disease and when used in combination with transient elastography for CFLD diagnosis, the diagnostic sensitivity and negative prediction was considerably improved. Thereby, TIMP-4 holds the potential to be a complimentary serum marker that facilitates the accurate non-invasive assessment of CFLD.

Endoglin is a TGF-β co-receptor that is involved in the regulation of the activity of TGF-β signaling, a key cytokine for hepatic fibrogenesis. Apart from endothelial cells, Endoglin is expressed on a variety of profibrogenic cell populations such as mesangial cells, scleroderma and cardiac fibroblasts and hepatic stellate cells (HSC) [45], [46], [47], [48]. A recent study has shown that Endoglin expression is increased in transdifferentiating hepatic stellate cells *in vitro* and in models of liver fibrosis in *vivo* [49]. As mechanism of action, Endoglin interacts with and is phosphorylated by TβRII and it has been shown *in vitro* that overexpression of Endoglin leads to TGF-β1-driven Smad1/5 phosphorylation and α-smooth muscle actin expression in a HSC, thereby promoting HSC activation [49]. First reports evaluated circulating Endoglin levels in patients with liver disease of diverse etiologies [50], [51]. Here, high Endoglin levels showed a significant association with the severity of chronic liver disease [50], [51], suggesting an active role for Endoglin in the fibrotic process.

In this report, we observed a significantly increased Endoglin expression in cystic fibrosis associated liver disease. Similar to our observations for TIMP-4, Endoglin serum levels were highest in HCV patients with complete liver cirrhosis, suggesting a close relation to hepatic staging. Likewise, Endoglin exhibited a high accuracy for the detection of CFLD and increased the diagnostic sensitivity and negative prediction considerably when used in combination with transient elastography. Thereby, our study identifies Endoglin as a further promising serum marker with the potential to improve the accurate non-invasive assessment of CFLD.

Hepatocyte growth factor is a mesenchyme-derived growth factor mainly produced by monocytes and hepatocytes. Although originally believed to be a liver specific mitogen [52], HGF is now recognized not only as a potent mitogenic growth factor on hepatocytes, but rather as a pleiotropic factor that exhibits mitogenic, motogenic, morphogenic, and antiapoptotic activities on various cell types. In the liver, HGF is produced by nonparenchymal cells, such as Kupffer cells, sinusoidal endothelial cells (SECs), and HSCs and the degree of liver HGF expression correlates with serum HGF levels [53]. The clinical significance of serum HGF levels has been assessed in acute hepatic failure and chronic liver diseases. In this respect it has been shown that HGF serum levels correlate with hepatic fibrosis and might serve as a prognostic maker in acute liver failure [54], [55], [56], [57]. Although the proteome screening within this report showed a relatively high abundance in patients with CFLD compared to those without, we were not able to verify this result in ELISA quantifications within the whole CF cohort. It might be speculated that one of the reasons for this might be due to the pleiotropic functions and lack of liver specificity of HGF as discussed above.

Pentraxins comprise a superfamily of evolutionary well-conserved proteins that are characterized by the pentraxin domains as a characteristic structural motif [58]. The short PTXs CrP and serum amyloid P are produced in the liver in response to inflammatory cytokines and therefore are classical acute-phase proteins. PTX3, as a prototypic member of the long pentraxin family, is induced primary upon inflammatory stimuli in a variety of cells, including monocytes/macrophages, dendritic cells, endothelial cells, vascular smooth muscle cells, fibroblasts, and adipocytes [59]. Due to the prominent role in the crossroad between innate immunity, ECM deposition and vascular biology [59], PTX3 serum levels have been assessed in a variety of diseases including vasculitis [60], myocardial infarction [61], [62] and systemic inflammation [63]. Importantly, serum PTX3 levels have been shown to be increased in patients with non-alcoholic steatohepatitis (NASH) and also to correlate with the histological staging in NASH [64]. As a rather unexpected result, we observed a significantly decreased expression of PTX3 in patients with CFLD rather an increase in our study. Similarly to HGF, it might well be that these discrepancies are due to the multiple functions of PTX3 on the one hand and also the different etiologies of liver disease, i.e. non-focally distributed NASH compared to focally distributed CFLD, on the other hand.

In conclusion, we used a proteome profiling based approach to identify serum markers of CFLD within the current study. Our results are the first to identify TIMP-4 and Endoglin as novel serum markers that can accurately diagnose CFLD. Further, their determination may confirm and enhance the diagnostic accuracy of TE for the detection of CFLD.

## Supporting Information

Figure S1 **Concentrations of serum biomarkers in relation to the severity of CF lung disease.** CF patients were stratified into those with a forced expiratory volume in one second (FEV1) below and above 70%, with a vital capacity (VC) of below and above 80%, and with a ratio between FEV1 and VC below and above 70% (FEV1/VC), all of which serve as established indicators of the severity CF lung disease. Neither TIMP-4 nor Endoglin or Pentraxin-3 differed in patients with and without impairment of lung function as assessed by FEV1, VC and FEV1/VC.
(TIF)

Figure S2 **Concentrations of serum biomarkers in CF patients with and without pancreatic insufficiency.** CF patients were stratified into those with (PI) and without pancreatic insufficiency (no PI). Neither TIMP-4 nor Endoglin or Pentraxin-3 differed in patients with and without pancreatic insufficiency.
(TIF)

Figure S3 **Liver stiffness and concentrations of serum biomarkers in CF patients with and without treatment of UDCA.** CF patients were stratified into those with (UDC) and without existing therapy with Ursodeoxycholic acid (no UDCA). While patients with UDCA exhibited a slightly increased liver stiffness, neither TIMP-4 nor Endolgin or Pentraxin-3 differed in patients with and without existing UDCA therapy.
(TIF)
