# Supplementary figures and images for: Serum Proteome Profiling Identifies Novel and Powerful Markers of Cystic Fibrosis Liver Disease
Source: PLoS One. 2013 Mar 14;8(3):e58955. doi: 10.1371/journal.pone.0058955 (PMC3597583; doi:10.1371/journal.pone.0058955)

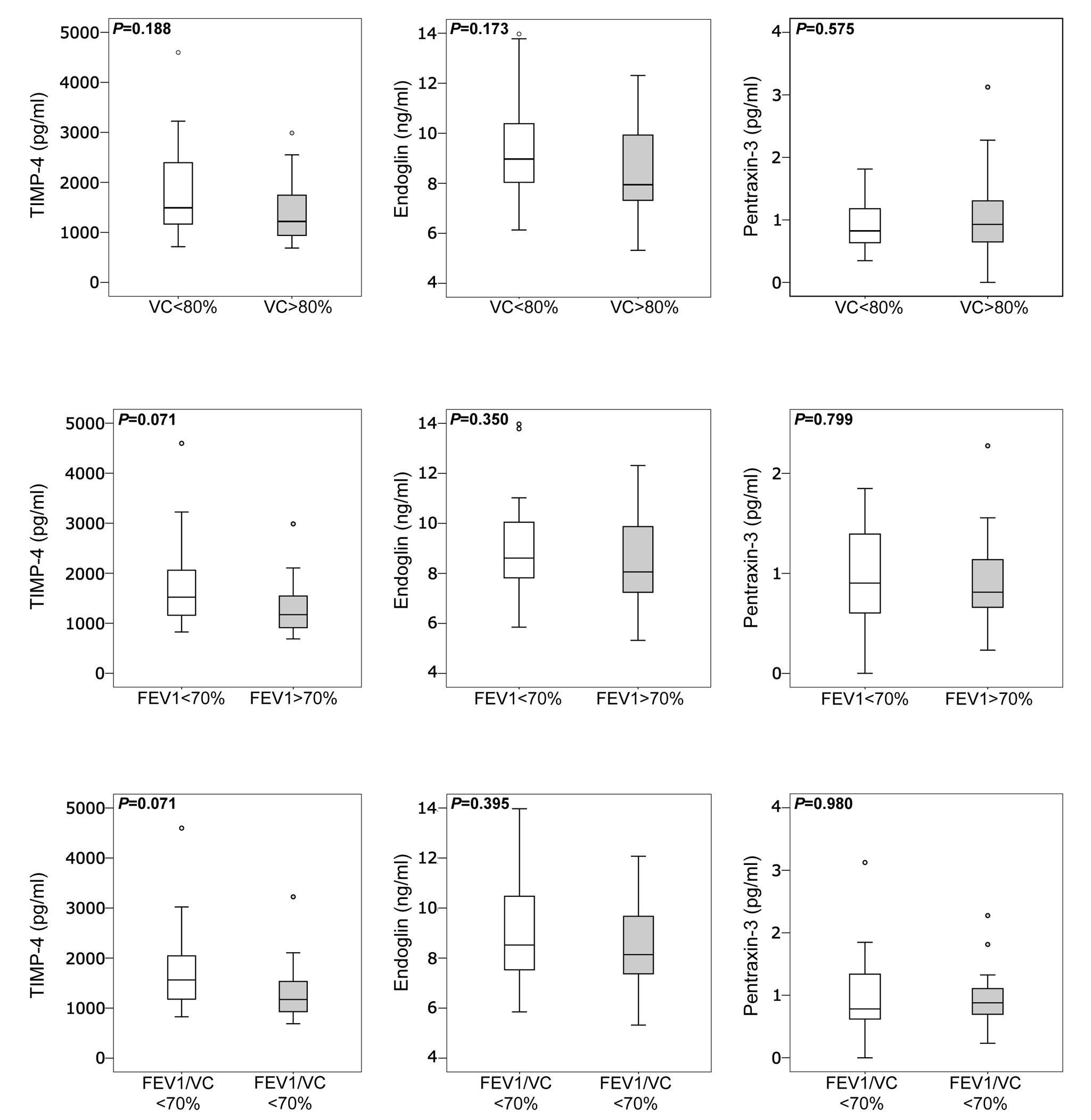

Supplement: Figure S1 — Concentrations of serum biomarkers in relation to the severity of CF lung disease. CF patients were stratified into those with a forced expiratory volume in one second (FEV1) below and above 70%, with a vital capacity (VC) of below and above 80%, and with a ratio between FEV1 and VC below and above 70% (FEV1/VC), all of which serve as established indicators of the severity CF lung disease. Neither TIMP-4 nor Endoglin or Pentraxin-3 differed in patients with and without impairment of lung function as assessed by FEV1, VC and FEV1/VC. (TIF) [file pone.0058955.s001.tif]

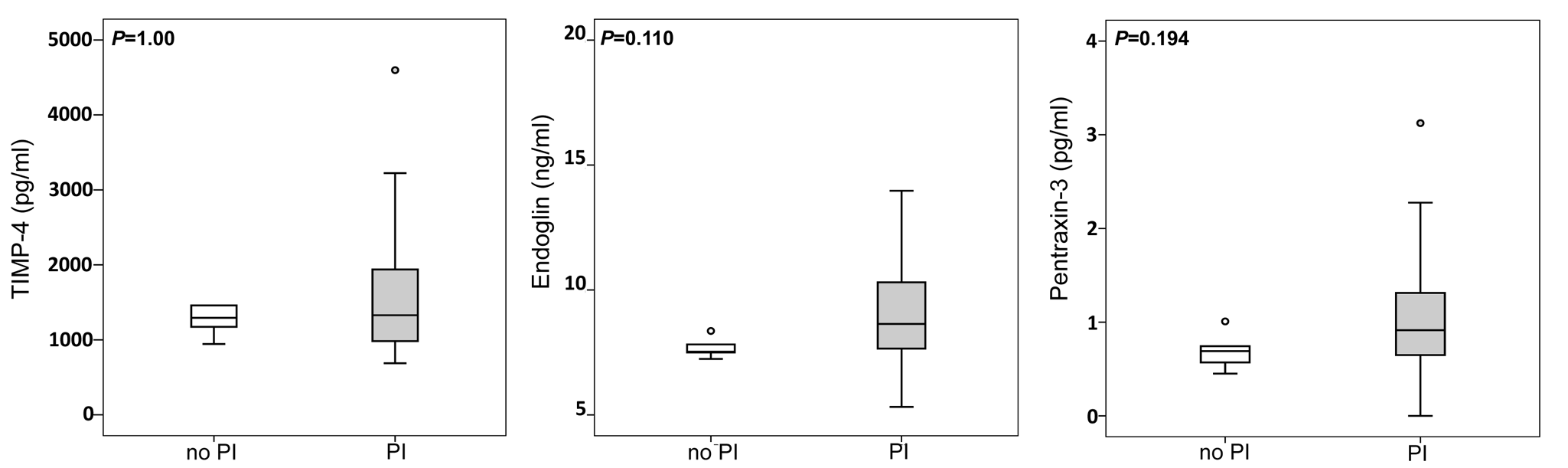

Supplement: Figure S2 — Concentrations of serum biomarkers in CF patients with and without pancreatic insufficiency. CF patients were stratified into those with (PI) and without pancreatic insufficiency (no PI). Neither TIMP-4 nor Endoglin or Pentraxin-3 differed in patients with and without pancreatic insufficiency. (TIF) [file pone.0058955.s002.tif]

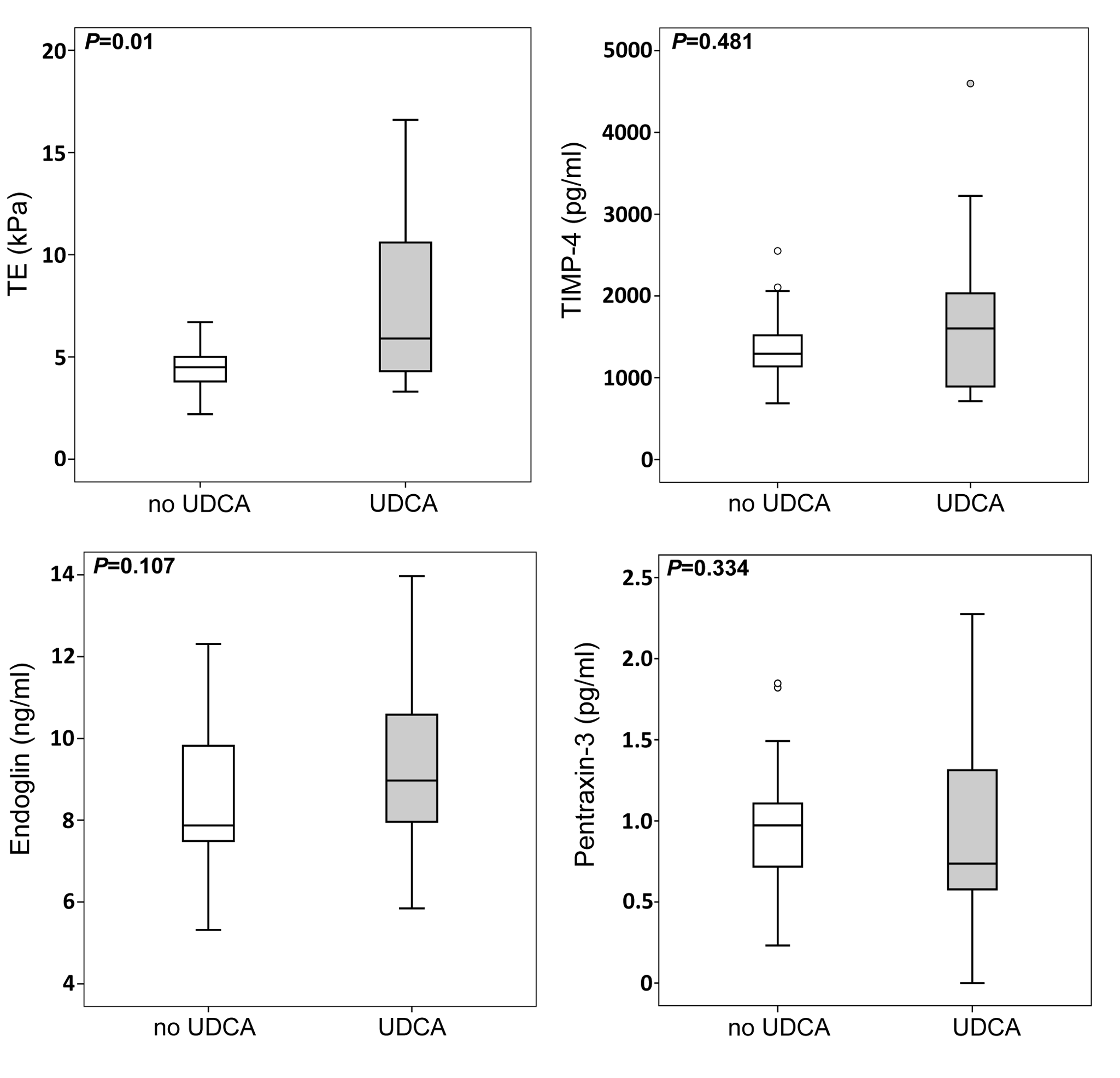

Supplement: Figure S3 — Liver stiffness and concentrations of serum biomarkers in CF patients with and without treatment of UDCA. CF patients were stratified into those with (UDC) and without existing therapy with Ursodeoxycholic acid (no UDCA). While patients with UDCA exhibited a slightly increased liver stiffness, neither TIMP-4 nor Endolgin or Pentraxin-3 differed in patients with and without existing UDCA therapy. (TIF) [file pone.0058955.s003.tif]
